# How Shall We Protect Our Girls?

**Published:** 1914-04

**Authors:** 


### How Shall We Protect Our Girls?

This burning question of the day, when the country has been aroused to fever heat by the campaign against the white slave traffic, with its accompanying revelations of the dangers and snares that encompass the footsteps of young girls, particularly those who have been forced into the business and labor worlds, has been answered by I. D. Steinhardt, M. D., in his book, "Ten Sex Talks to Girls Fourteen Years and Older." Price, $1.00. J. B. Lippincott Company, Publishers, Philadelphia.

"Forewarned is forearmed," is the motto adapted for this thoroughly good book. Its mission is delicately performed, with a good man's reverence for lofty and true womanhood. The doctor will find it invaluable when called upon to instruct his young patient in proper protection of her health; the mother and teacher can find no better means to employ for the instruction of the young girl in what it is vitally necessary she should know.

Teach the girls to protect themselves! should be the slogan of every mothers' club, teachers' association, the warning of the family physician, the battle cry of all the societies and individuals struggling for social betterment and uplift. The girl who has read this little book cannot become the innocent victim of the unscrupulous, nor can she make those blunders that fill hospitals, sanitariums and insane asylums with the unhappy wrecks of womanhood whose sins were merely ignorance.

The ten talks to girls that make up the book were delivered in their original form before various societies, and won such unqualified praise that the lecture rooms were unable to hold all who came to hear them. First published in the *New York Medical Journal,* the demand for copies of the numbers containing the lectures at once outran the supply. Dr. Steinhardt revised them, and enlarged them somewhat before offering them in book form, so that their usefulness might be greatly increased with the larger audience addressed.

The young wife and mother are addressed in several chapters in wise and helpful discussion of her duty and responsibility in the home and to her husband and children. Nothing more judicious could be written on these topics, and the doctor seeks to inspire his reader with his own high ideals and noble principles, in addition to the practical and scientific aspects of his articles. We would urge that the book be read in every home, and by all those interested in the welfare and protection of the young, and the progress of human society.

This book has the unqualified endorsement of Dr. Rachelle S. Yarros, chairman of social hygiene of the General Federation of Women's Clubs, who says:

"The study of physiology and hygiene of sex is bound to deepen our reverence for the wonderful processes of nature and to intensify our desire to make the best of our faculties and carefully fit ourselves for the noblest part in life."

We heartily commend the book to all progressive mothers.

---

The error is often made by capable Roentgenologists of mistaking the normal bone grooves of meningeal arteries for lines of skull fracture. Familiarity with the location of these grooves and comparative radiographs of the opposite side will obviate such an error.—*American Journal of Surgery.*

---

It is worth remembering that in Hodgkin's disease the glandular enlargements may be confined for a long time (even a year or more) to one side of the neck. In clinically differentiating this chronic, localized adenopathy from that of tuberculosis, absence of softening and of fusion of the glands, daily marked elevations of temperature, increasing anemia, and enlargement of the spleen, some or all of which signs and symptoms are usually present, are fairly diagnostic. A negative von Pirquet reaction, and a thick, doughy, pasty-appearing skin may also help the diagnosis.—*American Journal of Surgery.*

